# Artificial intelligence for chordee measurement in hypospadias surgery: current evidence and a blinded expert-panel validation framework

**DOI:** 10.3389/fped.2026.1895401

**Published:** 2026-07-28

**Authors:** Yusuf Atakan Baltrak, Hasan Deliaga

**Affiliations:** 1 Department of Pediatric Urology, Adana City Training and Research Hospital, Adana, Türkiye; 2 Department of Pediatric Urology, Bursa Yuksek Ihtisas Training and Research Hospital, University of Health Sciences, Bursa, Türkiye

**Keywords:** artificial intelligence, chordee, clinical validation, hypospadias, intraoperative imaging, machine learning, pediatric urology, penile curvature

## Abstract

Hypospadias-associated ventral penile curvature, commonly referred to as chordee, is a key intraoperative finding that can influence the choice of straightening technique and the need for urethral plate preservation or transection. In routine surgery, curvature is usually estimated visually during artificial erection testing, but this approach is subjective and may vary according to surgeon experience, viewing angle, erection quality, penile rotation, and image perspective. Artificial intelligence and computer vision methods may provide a more objective and reproducible way to measure curvature from standardized operative images. Early studies have used object detection, segmentation, landmark detection, Hough-transform methods, circle fitting, and geometric angle calculation. Reported technical performance is promising, particularly in controlled model-based datasets, but the clinical evidence remains limited because real intraoperative images are heterogeneous and because reference standards vary across studies. This Review summarizes the current evidence for artificial intelligence-based penile curvature measurement in hypospadias surgery and proposes a practical validation framework for future clinical studies. The most important next step is not simply improving model accuracy, but testing locked models on standardized real intraoperative images using patient-level data separation, blinded expert-panel measurements, threshold-focused analysis around 30 degrees, and direct comparison with the operating surgeon's live estimate. At present, artificial intelligence should be considered a measurement aid for documentation, quality improvement, and decision support, not a replacement for expert surgical judgment.

## Introduction

Hypospadias is one of the most common congenital anomalies of the male external genitalia. Ventral penile curvature is an important component of the deformity in many patients and may influence the choice of operative strategy, the need for additional straightening, and the expected functional and cosmetic outcome (1, 2).

During hypospadias repair, curvature is commonly assessed after penile degloving and artificial erection testing. The surgeon estimates the angle of curvature and decides whether degloving alone is sufficient or whether dorsal plication, ventral lengthening, urethral plate transection, or a staged strategy should be considered (1, 2). A threshold near 30 degrees is often treated as clinically important because it may change the operative plan, particularly in proximal hypospadias or in patients with persistent curvature after degloving (1, 3).

Despite its importance, intraoperative curvature assessment remains subjective. Visual estimation may be affected by surgeon experience, viewing angle, erection quality, penile rotation, operative exposure, lighting, image focus, and the morphology of the curvature. A recent global survey showed substantial variation in how hypospadias-associated penile curvature is assessed and managed, with visual estimation remaining common despite being perceived as less reliable than objective measurement tools (2).

Artificial intelligence (AI) and computer vision methods have recently been applied to penile curvature measurement. These approaches generally aim to identify the penile region, segment the penile shaft, detect key landmarks or the centerline, and calculate the curvature angle automatically from two-dimensional images (4–9). The goal is not to replace surgical judgment but to improve documentation, reduce measurement variability, and support multicenter research using a more reproducible measurement standard.

The clinical translation of AI-based curvature measurement requires more than a low average error in controlled images. A clinically useful model must perform reliably in real intraoperative conditions, avoid unsafe errors near the 30-degree threshold, and be validated against a reference standard that is less subjective than a single surgeon's live estimate. This Review summarizes the current evidence and proposes a practical validation framework for future studies in pediatric urology.

## Approach to this review

This article is a focused Review, not a PRISMA-compliant systematic review. It summarizes the current published and citable evidence on AI-based or automated image-based penile curvature measurement and integrates these findings into a proposed clinical validation framework. Because formal database exports, duplicate removal, and independent screening were not performed, the article should not be presented as a systematic review or scoping review.

The Review focuses on studies that evaluated chordee or penile curvature in the context of hypospadias, congenital penile curvature, or related pediatric penile curvature assessment; used AI, deep learning, computer vision, or automated image analysis; and reported quantitative or clinically interpretable performance metrics. Related work on semiautomated image-based angle estimation, prior scoping work on automated penile curvature assessment, and reporting standards for AI/diagnostic studies is discussed where it informs clinical validation (10–14).

## Why objective chordee measurement matters

The degree of penile curvature can directly influence operative decision-making. Mild curvature may improve after degloving alone, whereas persistent moderate or severe curvature may require additional straightening maneuvers. In borderline cases, a small difference in angle estimation may alter the choice between preserving and transecting the urethral plate or between single-stage and staged repair.

Subjective measurement also limits research. If centers use different visual thresholds or different photographic techniques, outcomes cannot be compared reliably. A reproducible measurement pipeline could support standardized operative documentation, objective reporting in clinical studies, and better comparison of surgical techniques across institutions.

Objective measurement does not remove the need for clinical interpretation. Curvature angle is only one component of the operative phenotype. Urethral plate quality, glans configuration, ventral tissue deficiency, corpus disproportion, penile torsion, skin availability, and tissue response after degloving all remain essential in surgical planning. Therefore, AI-based measurement should be framed as an adjunct to expert assessment rather than an autonomous surgical decision system.

## Current AI and computer vision approaches

Published AI-based pipelines generally include three or four technical steps: penile region localization, shaft segmentation, landmark or curve modeling, and final angle calculation. Object detection methods such as YOLO have been used to crop the penile region. U-Net-based models have been used to segment the penile shaft. Landmark-based models, including HRNet-based approaches, have been used to identify key points defining the proximal and distal shaft axes. Other methods have used Hough-transform or circle-fit optimization to estimate curvature geometrically (4–9).

Early proof-of-concept studies used three-dimensional printed penile models with known curvature angles. These datasets are useful because the true curvature is controlled and different lighting, background, or viewing conditions can be simulated. However, model-based datasets do not reproduce the full complexity of surgery. Real operative images include tissue edema, blood, skin folds, dartos tissue, instrument shadows, variable erection pressure, nonstandard camera angles, and different image resolutions. Performance in a controlled model dataset can therefore overestimate performance in clinical practice.

Later work moved toward more clinically relevant image sources, including real two-dimensional intraoperative images. In a real-image study, a dataset of 421 images was annotated by human experts and used to train a pipeline that localized the penile region, segmented the shaft, predicted four key points, and calculated curvature automatically. The model achieved a mean absolute error of approximately 7.9 degrees, with most predictions within 10 degrees and a low frequency of crossing the 30-degree surgical threshold (8). This type of evidence is more clinically relevant than model-only testing because it reflects the variability of operative imaging.

A concise overview of the main AI-based penile curvature assessment studies is presented in Table 1. The central message is that automated measurement is technically feasible, but the field is still early. Datasets are small, methods are heterogeneous, reference standards vary, and only limited evidence is available from real intraoperative images.

**Table 1:** Summary of AI-based and automated penile curvature assessment studies.

Study	Image source	Method	Reference standard	Main contribution
Abbas et al. (4)	3D-printed penile models	YOLOv5, U-Net, Hough-transform-based angle estimation	Known model curvature	Early proof of concept for automated penile curvature quantification
Baray et al. (5)	3D-printed model images with limited real-patient images	YOLOv5, U-Net, HRNet	Known model angles and expert measurement	Multi-stage deep learning pipeline for curvature measurement
Abbas et al. (6)	3D-printed models assessed by clinicians	AI-based tool compared with urologist assessment	Manual visual, goniometer, mobile app, and AI assessment	Clinical user validation against common manual methods
Sathya et al. (7)	Real and synthetic images	U-Net segmentation and circle-fit optimization	Known or annotated ground truth	Automated curvature quantification requiring further clinical validation
Baray et al. (8)	Real 2D intraoperative images	YOLOv8, segmentation, modified HRNet	Human expert annotation	Most clinically relevant real-image validation study
Babu et al. (9)	Pediatric hypospadias curvature images	U-Net segmentation and curvature pipeline	Study-specific reference standard	Recent AI-assisted pipeline requiring external clinical validation

AI, artificial intelligence; HRNet, High-Resolution Network; U-Net, convolutional neural network architecture used for segmentation; YOLO, You Only Look Once object detection model.

## Clinical importance of the 30-degree threshold

For hypospadias surgery, mean absolute error alone is not sufficient. A model with a low average error may still be clinically unsafe if its largest errors occur near a decision threshold. Conversely, a model with moderate average error may be acceptable for documentation if it rarely changes classification across clinically meaningful thresholds.

Future studies should therefore include threshold-focused metrics. These should include sensitivity and specificity for curvature greater than 30 degrees, the proportion of cases incorrectly classified across the 30-degree threshold, and subgroup analysis of borderline cases. Reporting only Dice coefficient, segmentation accuracy, or mean angle error does not answer whether the model is safe for surgical decision support.

Threshold performance should be interpreted cautiously. The 30-degree threshold is a practical clinical reference, not an absolute biological boundary. It may be applied differently depending on hypospadias severity, urethral plate quality, surgical philosophy, and patient anatomy. AI should support more consistent measurement around this threshold, but it should not dictate the complete operative plan.

## Reference standard limitations

A major limitation of the current literature is the absence of a uniform reference standard. Known angles from three-dimensional printed models are precise, but they do not represent real surgery. Human expert measurement is clinically relevant, but it contains interobserver variability. The operating surgeon's live estimate is important because it reflects real-world decision-making, but it should not be treated as a gold standard because visual estimation is itself subjective (1, 2, 7).

A stronger reference standard would be the median digital measurement of three blinded pediatric urologists using anonymized standardized images. Each expert should measure independently and remain blinded to the operating surgeon's estimate, AI output, surgical technique, and the other reviewers' measurements. Cases with large disagreement, for example more than 10 degrees, should be flagged for sensitivity analysis rather than forced into a single unqualified reference label.

The operating surgeon's live estimate should still be collected. It is valuable as a real-world comparator because it is the measurement that currently drives decision-making. However, the primary validation question should be whether AI agrees with a blinded expert-panel reference standard and whether it provides equal or better consistency than the live estimate.

## Proposed clinical validation framework

**Figure 1:**
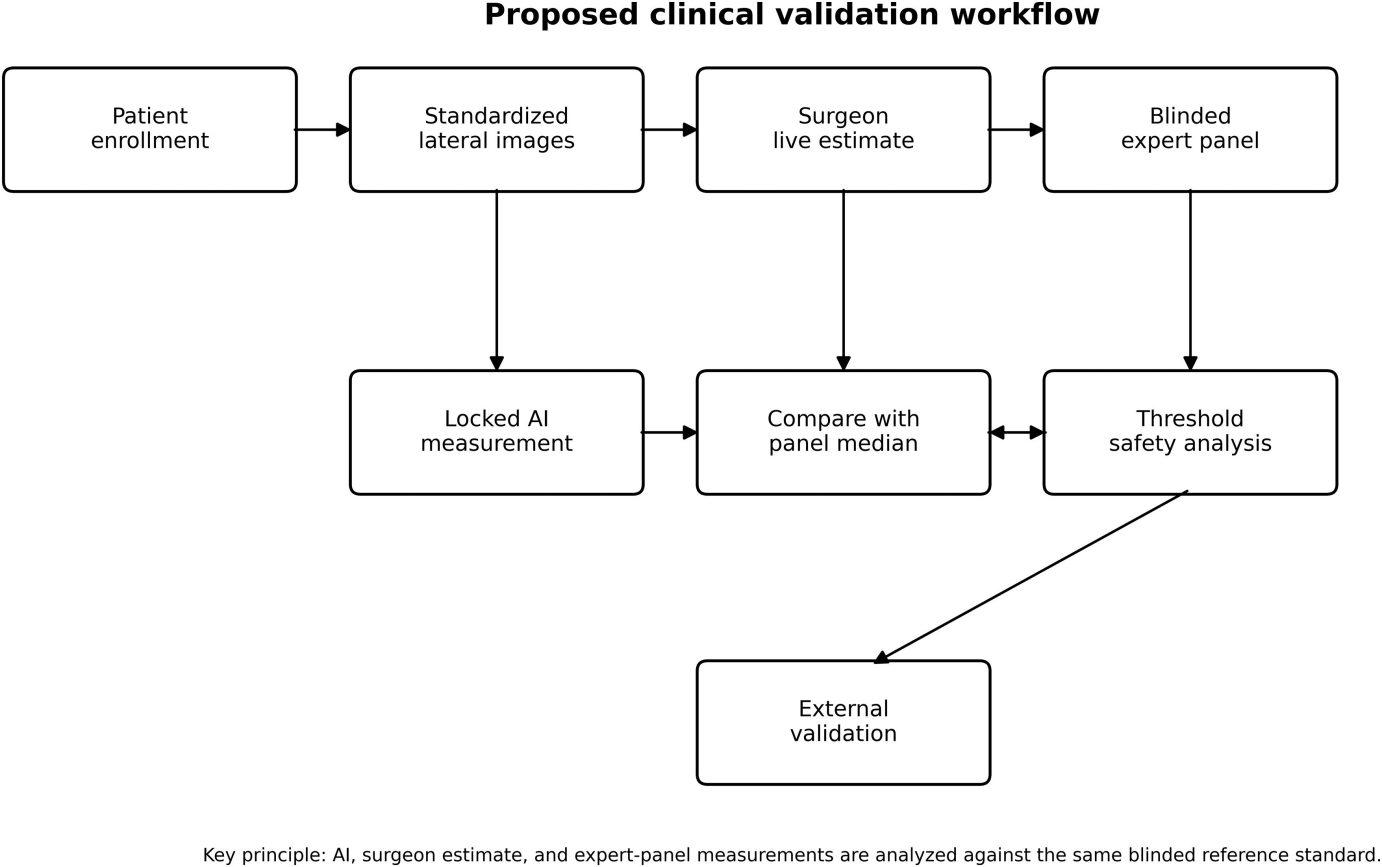
Proposed clinical validation workflow. The workflow includes patient enrollment, standardized intraoperative lateral imaging during artificial erection testing, the operating surgeon's live estimate, blinded three-expert digital measurement, locked AI-based measurement, comparison with the expert-panel median, threshold safety analysis, and external validation.

A practical validation workflow is summarized in Figure 1 and the recommended outcomes are listed in Table 2.

**Table 2:** Recommended outcomes for future clinical validation studies.

Domain	Recommended metric
Angle accuracy	Mean absolute error; root mean squared error
Agreement	Intraclass correlation coefficient; Bland-Altman bias and limits of agreement
Clinical threshold	Sensitivity and specificity for curvature greater than 30 degrees
Threshold safety	Incorrect crossing of the 30-degree threshold
Practical reliability	Model failure rate; image-quality subgroup performance
Human comparison	Surgeon estimate vs. expert-panel median; AI vs. expert-panel median
Generalizability	External validation; center-level performance

### Target population

Future studies should include boys undergoing primary hypospadias repair with artificial erection testing. The study population should be described by age, meatal location, hypospadias severity, prior hormonal stimulation, penile torsion, and whether curvature persists after degloving. Redo cases, severe torsion, disorders of sex development, and poor-quality images should either be excluded from the primary analysis or analyzed as prespecified subgroups.

Patient-level enrollment is essential. If multiple images are taken from the same patient, all images from that patient must remain in the same training, validation, or test partition. Image-level random splitting creates data leakage and can substantially overestimate model performance.

### Image acquisition

Images should be captured after complete degloving and during artificial erection testing, before any curvature correction. A true lateral view should be used whenever possible, with the full penile shaft and glans visible, without patient-identifying features. At least three images per patient should be acquired to allow quality assessment and sensitivity analysis.

Image quality should be graded before AI analysis. Minimum criteria should include adequate focus, adequate exposure, full shaft visualization, absence of major obstruction by instruments or hands, and acceptable lateral alignment. The model failure rate should be reported, including the reasons for failure.

### Expert-panel reference standard

Three pediatric urologists should independently measure the curvature angle on anonymized images using a prespecified digital method. The median of the three measurements should be used as the primary reference standard. Interobserver agreement should be reported using intraclass correlation coefficients and Bland-Altman analysis where appropriate.

**Figure 2:**
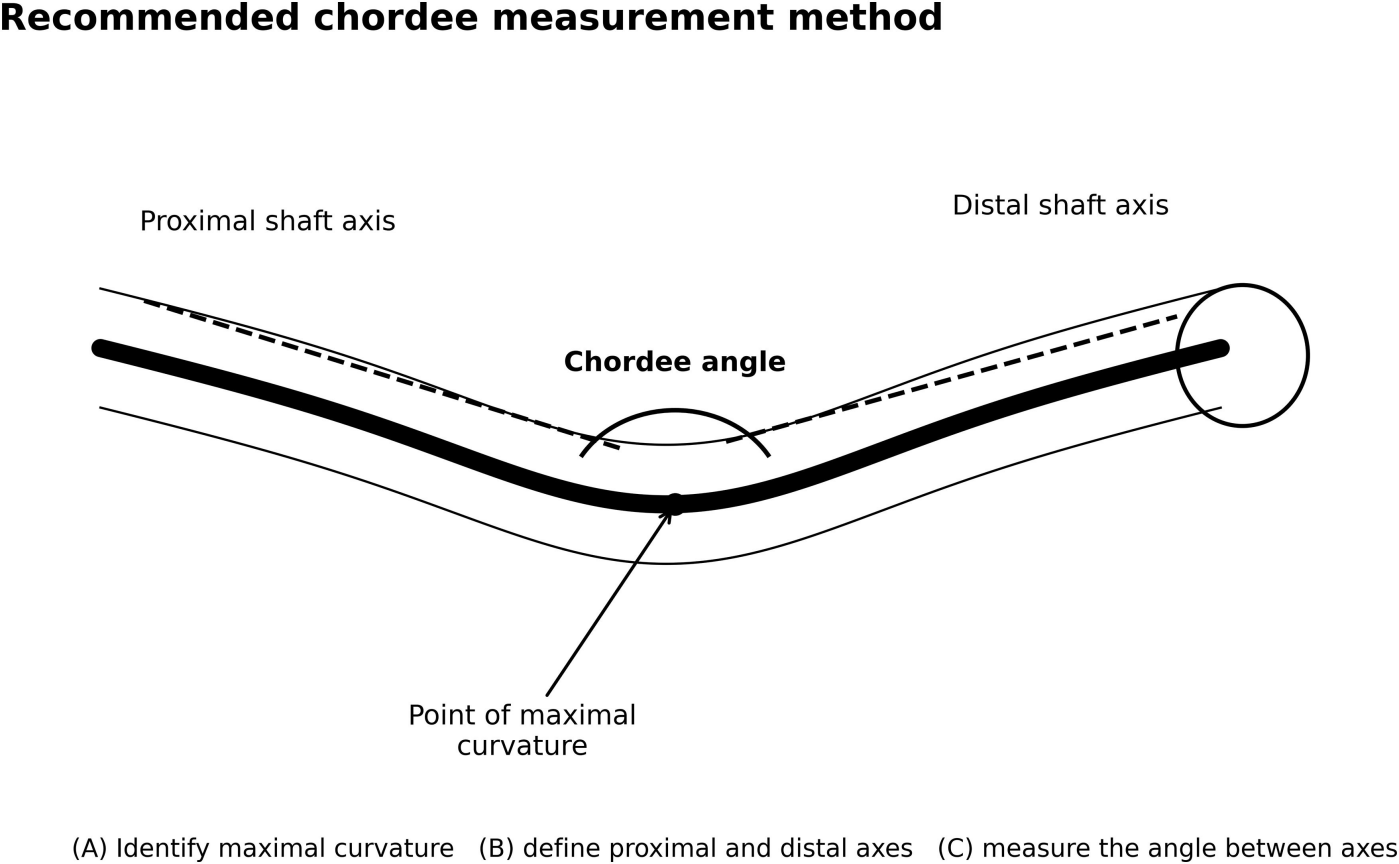
Recommended chordee measurement method. **(A)** Identification of the point of maximal curvature. **(B)** Definition of the proximal and distal shaft axes. **(C)** Measurement of the chordee angle between the two axes.

The measurement method should be standardized. For hinge-type curvature, the angle between the proximal and distal shaft axes should be measured. For long arc-type curvature, the point of maximal curvature and the proximal and distal shaft axes should be defined in a reproducible way. Figure 2 illustrates the recommended conceptual approach.

### Locked AI model testing

The AI model should be locked before final testing. Model architecture, training strategy, input resolution, preprocessing, augmentation, hyperparameters, software versions, and random seed should be documented. The final test set should not be used for model selection.

External validation is needed before clinical implementation. A model trained on images from one center, camera system, or surgical team may not generalize to another. Multicenter validation should include center-level performance reporting and subgroup analysis according to image quality and hypospadias severity.

### Recommended outcomes

Clinical validation should report mean absolute error, root mean squared error, intraclass correlation coefficient, Bland-Altman bias and limits of agreement, the proportion of predictions within 5 and 10 degrees, sensitivity and specificity for curvature greater than 30 degrees, incorrect crossing of the 30-degree threshold, and model failure rate.

The surgeon's live estimate should be analyzed as a secondary comparator against the same expert-panel median. This allows the study to determine whether AI adds value beyond current practice rather than simply reproducing a subjective estimate.

## Reporting and implementation considerations

Future studies should provide enough technical and clinical detail to allow replication. Reports should follow principles from diagnostic accuracy and AI reporting guidance, including STARD, TRIPOD + AI, and DECIDE-AI when applicable (11–13). At minimum, authors should describe the image protocol, patient-level data split, annotation process, reference standard, model architecture, validation strategy, failure cases, and threshold performance.

Implementation also requires attention to workflow. A useful system must provide results quickly enough to support intraoperative documentation, should identify low-quality or nonanalyzable images, and should display uncertainty when confidence is low. It should not provide an unqualified angle when the image is not appropriate for analysis.

Ethical and privacy issues are also relevant. Operative images must be de-identified, stored securely, and used according to institutional and national regulations. If AI outputs are used prospectively during surgery, investigators should specify whether surgeons are blinded to the AI output, whether the output can change the operative plan, and how adverse or discordant cases will be monitored.

## Limitations of the current evidence

The evidence base remains limited. The number of original AI-based curvature measurement studies is small, and the studies differ in image source, dataset type, model architecture, validation strategy, and reference standard. Controlled model images are useful for proof of concept but may overestimate real-world performance. Real intraoperative datasets remain relatively small and require broader external validation.

Another limitation is that most current methods use two-dimensional images. A single lateral image can estimate sagittal ventral curvature but cannot fully evaluate torsion, lateral curvature, multiplanar deformity, asymmetric tissue deficiency, or the dynamic response of tissues during degloving and straightening. These factors remain within the domain of expert surgical judgment.

Finally, most studies report measurement performance rather than clinical outcomes. It remains unknown whether AI-assisted measurement changes surgical decision-making, reduces residual curvature, improves long-term functional outcomes, or standardizes multicenter research reporting. These are important questions for future prospective studies.

## Discussion

AI-based chordee measurement is a promising development for pediatric urology because it addresses a specific and clinically meaningful source of subjectivity. The field has moved from controlled model-based proof of concept toward real intraoperative image analysis, and current results suggest that automated angle measurement can approach human expert performance in selected settings (4–9, 14).

The main barrier to clinical translation is not whether AI can calculate an angle from an image. The more important question is whether it can provide reliable, explainable, and threshold-safe measurements in the operating room. Future studies should therefore prioritize standardized image capture, patient-level separation, locked-model testing, blinded expert-panel reference standards, and external validation.

The proposed framework is intentionally practical. It allows AI to be tested against the current clinical workflow while avoiding the methodological weakness of treating the operating surgeon's visual estimate as an unquestioned gold standard. It also emphasizes threshold safety, because clinical harm would be more likely to arise from misclassification around a treatment-changing threshold than from small average errors in clearly mild or clearly severe cases.

At this stage, AI should be described as a decision-support and documentation tool. It may help surgeons record curvature more consistently, support training, improve research comparability, and identify borderline cases that need careful review. It should not be marketed or interpreted as a system that independently determines the hypospadias repair strategy.

## Conclusion

AI-based measurement of penile curvature is technically feasible and clinically promising for hypospadias surgery. Current evidence supports its potential role in objective documentation and decision support, especially near clinically important thresholds. However, the evidence is not yet strong enough to support independent clinical decision-making.

Future studies should use standardized real intraoperative images, patient-level data separation, locked AI models, blinded three-expert reference standards, direct comparison with the surgeon's live estimate, threshold-focused performance metrics, and external validation. Until such evidence is available, AI should be used as a measurement aid and research standardization tool, not as a replacement for expert pediatric urologic judgment.
